# Supplementary material for: The genome sequence of the Ruddy Flat-body, Agonopterix subpropinquella (Stainton, 1849)
Source: Wellcome Open Res. 2023 Oct 23;8:487. [Version 1] doi: 10.12688/wellcomeopenres.20170.1 (PMC11074695; doi:10.12688/wellcomeopenres.20170.1)
Supplement: Peer review history

# Reviewer report: Reviewer response for version 1

**DOI:** 10.21956/wellcomeopenres.22333.r81324

**Published:** 2024-05-15

**Authors:** Wei Zhang

**Affiliations:** 1 Peking University, Beijing, Beijing, China

This manuscript reports a chromosome-level genome assembly of the Rubby Flat-body ( *Agonopterix subpropinquella*) with the Z chromosome assembled. This genome assembly has an N50 value of 25.1 Mb.  It also has considerable BUSCO statistics and annotations. The methods section is well written. I think the overall quality of this genome assembly is valid and will benefit relevant studies. Therefore, I support the acceptance of the manuscript to be indexed.

Are sufficient details of methods and materials provided to allow replication by others?

Yes

Is the rationale for creating the dataset(s) clearly described?

Yes

Are the datasets clearly presented in a useable and accessible format?

Yes

Are the protocols appropriate and is the work technically sound?

Yes

Reviewer Expertise:

butterfly evolutionary genomics

I confirm that I have read this submission and believe that I have an appropriate level of expertise to confirm that it is of an acceptable scientific standard.

# Reviewer report: Reviewer response for version 1

**DOI:** 10.21956/wellcomeopenres.22333.r75821

**Published:** 2024-05-06

**Authors:** Naciye Sena Çağatay

**Affiliations:** 1 University of Liverpool, Liverpool, England, UK

The complete genome of the Ruddy Flat-body *Agonopterix subpropinquella* was assembled with Boyes and Hammond using PacBio HiFi sequencing, 10X Genomics and Hi-C Illumina sequencing techniques. The analysis were explained very well in each method. The genome assembly is of high quality and the figures shown are informative. I have a minor revision below.

 Background

 Can you check these words.

 Like many species in its genus, the imago is ‘’image’’

 Eggs are lain in May on knapweeds “laid”

 Q1:

 I was just wondering, there is no information about morphological identification. How do you decide this species? Why are you using male sample?

Are sufficient details of methods and materials provided to allow replication by others?

Yes

Is the rationale for creating the dataset(s) clearly described?

Yes

Are the datasets clearly presented in a useable and accessible format?

Yes

Are the protocols appropriate and is the work technically sound?

Yes

Reviewer Expertise:

Molecular entomology

I confirm that I have read this submission and believe that I have an appropriate level of expertise to confirm that it is of an acceptable scientific standard.

# Reviewer report: Reviewer response for version 1

**DOI:** 10.21956/wellcomeopenres.22333.r75813

**Published:** 2024-03-27

**Authors:** Simo Njabulo Maduna

**Affiliations:** 1 Norwegian Institute of Bioeconomy Research, Svanvik, Norway

The near-complete genome of the Ruddy Flat-body *Agonopterix subpropinquella* was assembled by Boyes and Hammond through the utilization of BacBio HiFi sequencing, as well as 10X Genomics and Hi-C Illumina sequencing techniques. The authors present the initial and superior reference genome for the Ruddy Flat-body. Their manuscript is skilfully composed and includes an elaborate bioinformatics pipeline and succinct findings, accompanied by genome assembly metric benchmarks to facilitate comprehension. Below I present minor revisions.

 ABSTRACT

 Include a sentence on the sequencing strategy employed to generate the reported chromosomal-level genome assembly for the target species.

 BACKGROUND

 Kindly incorporate a sentence regarding the genome sizes of moths, accompanied by a few examples, to provide the reader with an understanding of the average genome size of moths.

 Change “…Like many species in its genus…” to “…Like many of its congeners…”

 Question: What was the rationale behind selecting a homogametic male for sequencing rather than a heterogametic female? The reviewer is merely inquisitive given that the research gap is now on the assembly of the W sex chromosome.

Are sufficient details of methods and materials provided to allow replication by others?

Yes

Is the rationale for creating the dataset(s) clearly described?

Yes

Are the datasets clearly presented in a useable and accessible format?

Yes

Are the protocols appropriate and is the work technically sound?

Yes

Reviewer Expertise:

Genome Assembly; Ecological Genomics; Conservation Genomics; Invasive Genomics; Population Genomics; Phylogenomics; Molecular Phylogenetics

I confirm that I have read this submission and believe that I have an appropriate level of expertise to confirm that it is of an acceptable scientific standard.
